# Diffusion kurtosis imaging: An efficient tool for evaluating age‐related changes in rat brains

**DOI:** 10.1002/brb3.2136

**Published:** 2021-09-24

**Authors:** Xue‐Fang Han, Zuo‐Jun Geng, Qing‐Feng Zhu, Zhen‐Hu Song, Huan‐Di Lv

**Affiliations:** ^1^ Department of Radiology the Second Hospital of Hebei Medical University Shijiazhuang Hebei Province P.R. China

**Keywords:** age‐related correlation analysis, brain aging, diffusion kurtosis imaging, magnetic resonance imaging, Sprague–Dawley rat

## Abstract

**Purpose:**

To evaluate and determine age‐related changes in rat brains by studying the diffusion kurtosis imaging results among different age groups of rats.

**Methods:**

Sprague–Dawley (*SD*) rats underwent conventional magnetic resonance imaging (MRI) and diffusion Kurtosis Imaging (DKI). Two diffusion values of mean kurtosis (MK) and kurtosis (K_⊥_) were measured and analyzed based on laterality, brain regions and age groups. The MK and K_⊥_ data were plotted against different age groups.

**Results:**

No laterality was found for the MK or K_⊥_ values in the cerebral cortex (CT), external capsule (EC), or caudate putamen (CPu) regions. In contrast, significant changes in these values were observed among different age groups. Changes of the MK and K_⊥_ values were significant in both hemispheres in the EC, the CT, and the CPu brain regions. The changes in the MK and K_⊥_ values showed a parabolic relationship with ages in all the brain regions.

**Conclusion:**

No laterality in the MK and K_⊥_ values was observed for the EC, CT, or CPu regions of the rat brain. Significant changes in MK and K_⊥_ values were both observed among different age groups, thus suggesting diffusion kurtosis imaging as an efficient tool for studying brain aging in rats.

## Graphical abstract

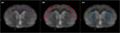

## 1 INTRODUCTION

Brain aging and related neural degenerative diseases have become a serious health issue with the continuing growth of the elder population (Ferreira & Busatto, 2013; Jagust et al., 2006; Thal et al., 2004). Therefore, an increasing amount attention has been drawn to research areas where new tools for investigating brain aging process are studied (Angelie et al., 2001; Ge et al., 2002; Lockhart & DeCarli, 2014); with the purpose of unravel its underlying mechanism and discover treatments that could effectively prevent and slow down the progression of brain aging. Development, maturity, and aging of brain tissue are extremely complex processes involving morphological and structural changes (Mukherjee et al., 2001). The development of noninvasive in vivo imaging techniques such as MRI (Courchesne et al., 2000; Gunning‐Dixon et al., 2009), diffusion tensor imaging (DTI; Assaf & Pasternak, 2008; Madden et al., 2009; Moseley, 2002) have greatly improved our ability in evaluating the structural and functional changes caused by brain aging. DTI is a new MRI imaging technique based on Gaussian distribution model to reflect the diffusion direction and diffusion volume of water molecules in tissues. It can quantitatively evaluate the anisotropy of the diffusion direction by measuring the apparent diffusion coefficients (ADC) in at least six independent directions. At present, the main quantitative indexes of DTI to describe anisotropy parameters are fractional anisotropy (FA), eigenvalue (λ1, λ2, λ3), and mean diffusivity (MD). Due to the assumption of Gaussian distribution, it has limitations in the application of cross fiber tissue. Recently, DKI has emerged as a powerful imaging technique as an extension DTI (Falangola et al., 2008; Steven et al., 2014). DKI aims to describe the non‐Gaussian aspects of water diffusion and that can more realistically and objectively reflect tissue microstructure.

In this study, in *vivo* DKI experiments were performed on normal *SD* rats to analyze the water diffusion values in different brain regions including CT, EC, and CPu and to reveal the relationship between the changes in these values and brain aging. Two diffusion values of mean kurtosis (MK) and kurtosis (K_⊥_) were measured and analyzed based on laterality, brain regions and age groups. MK corresponds to the apparent kurtosis coefficient averaged over all directions, just as the mean diffusivity corresponds to the diffusion coefficient averaged over all directions. The radial direction is the maximum limited direction for water molecular diffusion; thus, the K_⊥_ value is considered as its primary influencing factor. This study was designed to further explore the physiological and pathological changes related to normal aging of brain tissue by employing DKI technique and to lay the foundation for studies of age‐related degenerative diseases.

## 2 METHODS

### 2.1 Ethical statement

This study was approved by the Ethics Committee of the Second Hospital of Hebei Medical University.

### 2.2 Animal preparation

The animal experiments used a total of 40 male *SD* rats (all acquired at 9 weeks old) and were approved by Beijing Weitonglihua Laboratory Animal Technology. The animals were housed in standard polypropylene cages with wired‐net tops in a controlled room (temperature 21 ± 2℃, 12‐hr light‐dark cycle) and were allowed free access to a standard laboratory pellet diet and water throughout the experiments. The animals were subsequently assigned to one of four age groups of 3 months (M3, *N* = 10), 6 months (M6, *N* = 10), 10 months (M10, *N* = 10), and 13 months (M13, *N* = 10). The study received approval from the ethics committee of the Second Hospital of Hebei Medical University.

The inclusion and exclusion criteria were as follows: (a) no dysplasia, (b) no abnormal signals discovered by routine MRI examination, and (c) exclusion of unsatisfactory images by image postprocessing.

### 2.3 Data collection

Imaging was performed using a horizontal 3.0 Tesla MR system (GE HD750) equipped with a 50‐mm inner diameter coil. The animals were anesthetized using 10% chloral hydrate via intraperitoneal injection at 0.3 ml/100 g. Then, all animals underwent conventional MRI, including coronal T1WI and coronal, sagittal and axial T2WI and DKI. The acquisition parameters were as follows: coronal T1WI: TR/TE = 2,833.3/24 ms, data matrix = 256 × 192, number of excitations (NEX) = 3, FOV = 60 mm, slice thickness = 3 mm, slice gap = 0 mm, and angle = 111 degrees; coronal T2WI: TR/TE = 1500/25.5 ms, data matrix = 512 × 512, NEX = 10, FOV = 60 mm, slice thickness = 3 mm, slice gap = 0 mm, and angle = 111 degrees; sagittal T2WI: TR/TE = 1545/44.7 ms, data matrix = 512 × 512, NEX = 10, FOV = 60 mm, slice thickness = 3 mm, slice gap = 0 mm, and angle = 111 degrees; axial T2WI: TR/TE = 2303/46.8 ms, data matrix = 512 × 512, NEX = 10, FOV = 60 mm, slice thickness = 3 mm, slice gap = 0 mm, and angle = 111 degrees; and DKI pulse sequence: a subsaturation zone covering the respiratory tract to reduce image artifacts, a shim of 6 cm × 6 cm in size covering the entire brain, TR/TE = 2000/97.3 ms, data matrix = 64 × 64, FOV = 60 mm, slice thickness = 4 mm, slice gap = 0 mm, NEX = 2, number of diffusion‐encoding gradient directions = 25, number of *b*_0_ = 5, and *b*‐values (0, 1,000, and 2,000 s/mm^2^) applied along each direction. The voxel size was 0.9 mm × 0.9 mm × 4 mm. The voxels were anisotropic. The number of averages on the datasets was showed in the Table 1.

**TABLE 1:** The number of averages on the datasets of MK and K⊥values for the left and right CT, EC, and CPu

		3 months	6 months	10 months	13 months
MK	Left CT	0.771	0.867	0.839	0.440
	Right CT	0.753	0.819	0.842	0.461
	Left EC	0.685	0.713	0.670	0.403
	Right EC	0.664	0.696	0.692	0.417
	Left CPu	0.796	0.928	0.863	0.709
	Right CPu	0.777	0.900	0.854	0.670
K⊥	Left CT	0.714	0.833	0.805	0.508
	Right CT	0.694	0.810	0.787	0.545
	Left EC	0.617	0.644	0.646	0.482
	Right EC	0.638	0.649	0.659	0.490
	Left CPu	0.729	0.878	0.859	0.676
	Right CPu	0.752	0.895	0.847	0.648

Abbreviations: CT, cerebral cortex, EC, external capsule, CPu, caudate putamen.

### 2.4 Data analysis

**FIGURE 1:**
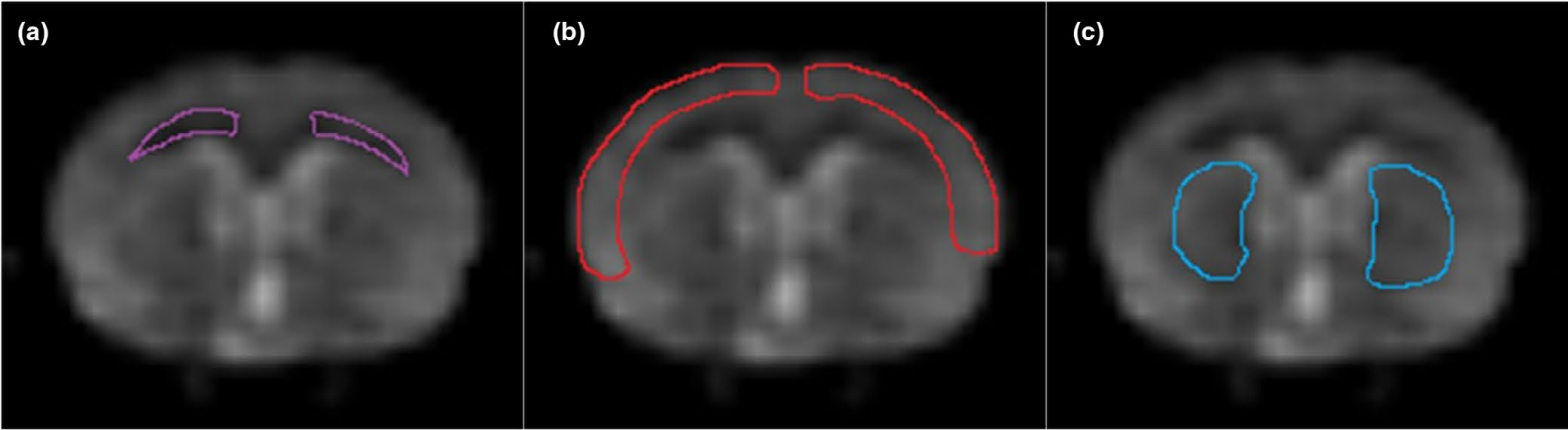
The results of regions of interest

Regions of interest (ROIs) were manually defined in a DKI map according to the anatomical landmarks identified from the standard rat brain atlasm (Paxinos & Watson, 2005), including three regions: the CT, EC, and CPu region. Two standard diffusion kurtosis metrics, MK and K_⊥_, were measured for each region. MK and K_⊥_ were measured again at one week and one month, and the average values were recorded as the corresponding diffusion values. The ROIs were defined as Figure 1.

Using SPSS 17.0, we evaluated the differences in diffusion values between the left and right side of the CT, EC, and CPu region using paired‐sample *t* tests. Additionally, we tested the significance of differences in diffusion values based on brain regions and age groups using one‐way ANOVA followed by the fisher's least significant difference test (LSD). Finally, we analyzed the relationship of different age groups with the diffusion values for each examined brain region and plotted these values against age groups.

## 3 RESULTS

The results of measurement and statistical analysis of the MK and K_⊥_ values for the left and right CT, EC, and CPu are shown in Table 2. The results from paired‐samples *t* tests indicated no laterality of the MK and K_⊥_ values was found for the CT, EC, or CPu brain regions (*p* < .05). Subsequently, the diffusion values were analyzed separately for each hemisphere to detect hemisphere‐specific differences.

**TABLE 2:** The results of measurement and statistical analysis of the MK and K_⊥_values for the left and right CT, EC, and CPu

Brain area	Month	MK				K⊥			
		Left	Right	*t*	*p*	Left	Right	*t*	*p*
CT	3	0.685 ± 0.064	0.664 ± 0.074	0.679	.514	0.617 ± 0.065	0.638 ± 0.059	−0.778	.456
	6	0.713 ± 0.174	0.696 ± 0.076	0.380	.713	0.644 ± 0.139	0.649 ± 0.126	−0.104	.919
	10	0.670 ± 0.092	0.692 ± 0.061	−1.059	.317	0.646 ± 0.099	0.659 ± 0.094	−0.381	.712
	13	0.403 ± 0.074	0.417 ± 0.135	−0.436	.673	0.482 ± 0.111	0.490 ± 0.140	−0.167	.871
EC	3	0.771 ± 0.076	0.753 ± 0.088	1.217	.255	0.714 ± 0.074	0.694 ± 0.095	0.492	.634
	6	0.867 ± 0.140	0.819 ± 0.115	1.614	.141	0.833 ± 0.092	0.810 ± 0.132	0.600	.564
	10	0.839 ± 0.096	0.842 ± 0.077	−0.089	.931	0.805 ± 0.105	0.787 ± 0.080	0.448	.665
	13	0.440 ± 0.102	0.461 ± 0.080	−0.854	.415	0.508 ± 0.102	0.545 ± 0.077	−1.717	.120
CPu	3	0.796 ± 0.105	0.777 ± 0.070	0.974	.355	0.729 ± 0.117	0.752 ± 0.118	−0.774	.459
	6	0.928 ± 0.152	0.900 ± 0.190	0.565	.586	0.878 ± 0.135	0.895 ± 0.191	−0.241	.815
	10	0.863 ± 0.054	0.854 ± 0.083	−0.255	.799	0.859 ± 0.104	0.847 ± 0.112	0.476	.645
	13	0.709 ± 0.125	0.670 ± 0.105	1.294	.228	0.676 ± 0.138	0.648 ± 0.124	0.750	.472

Abbreviations: CT, cerebral cortex, EC, external capsule, CPu, caudate putamen.

**FIGURE 2:**
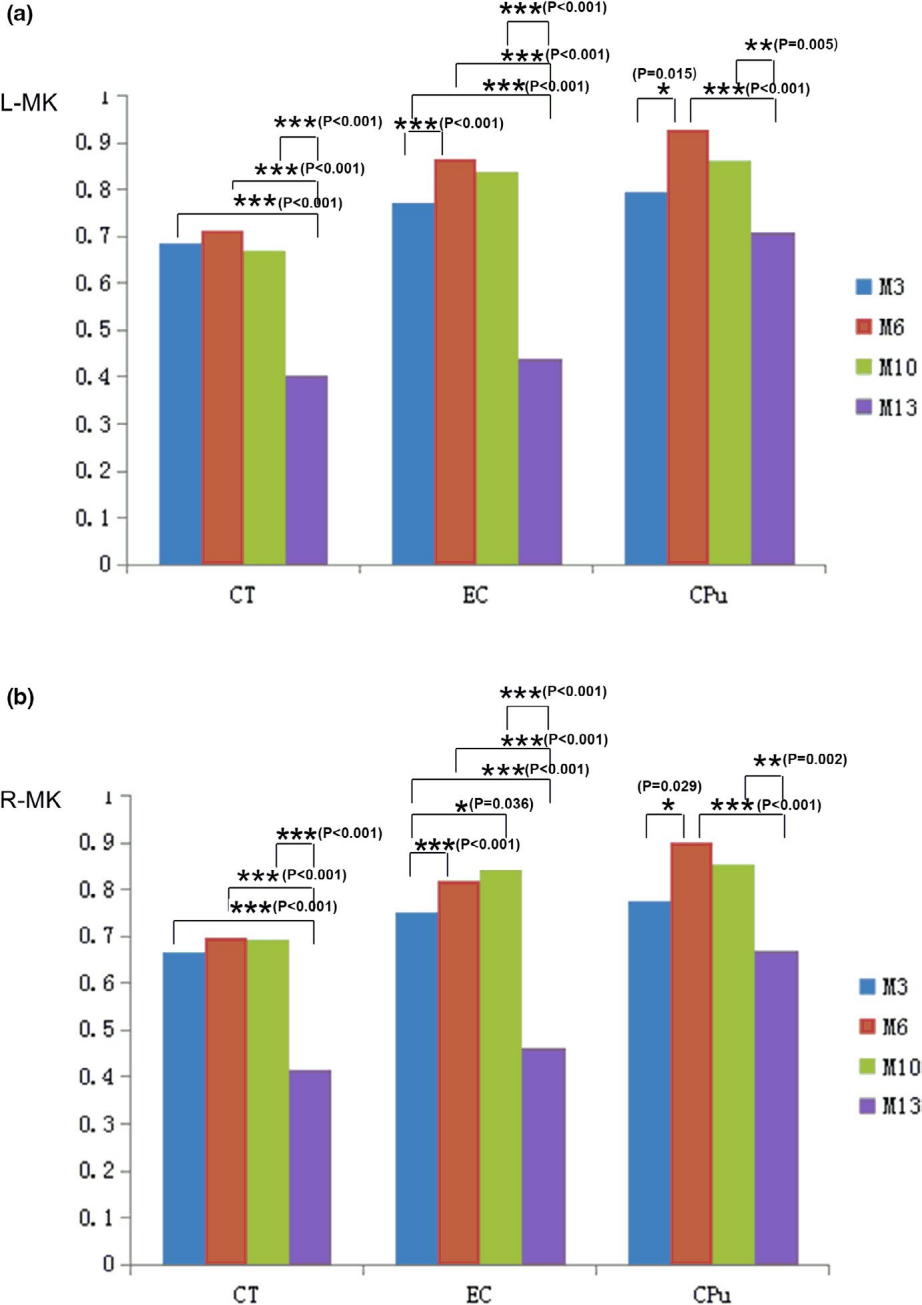
Comparison of the MK values for the CT, the EC, and the CPu among different age groups. *p < .05; **p < .01

The results of measurement and statistical analysis of the MK values in the CT, the EC and the CPu regions for four age groups are summarized at Figure 1. The results showed that the MK values at the both hemispheres of CT and EC regions vary significantly among different age groups. The MK values at the CPu region were significantly different between M3 and M6, between M6 and M13, and between M10 and M13. The significant differences among different group were showed in the Figure 2.

**FIGURE 3:**
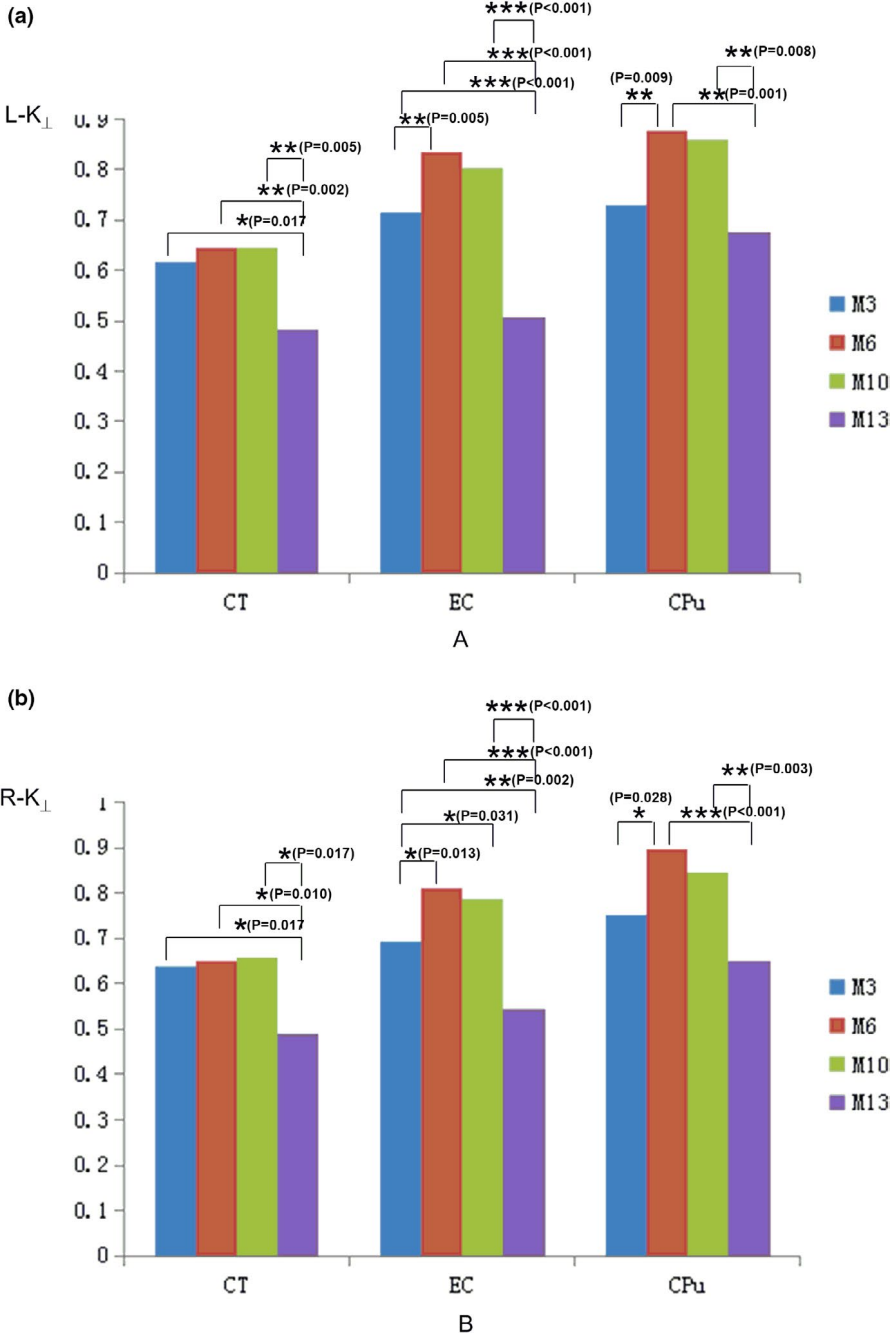
Comparison of the K_⊥_ values for the CT, the EC, and the CPu among different age groups. *p < .05; **p < .01

The results of measurement and statistical analysis of the K_⊥_ values in the CT, the EC, and the CPu regions for four age groups are summarized at Figure 2. The results showed that the K_⊥_ value at the CT region for M13 age group was significantly smaller than that for M3, M6, and M10 age groups. The K_⊥_ values at the EC and CPu regions in both hemispheres vary among all age groups. The significant differences among different group were showed in the Figure 3.

**FIGURE 4:**
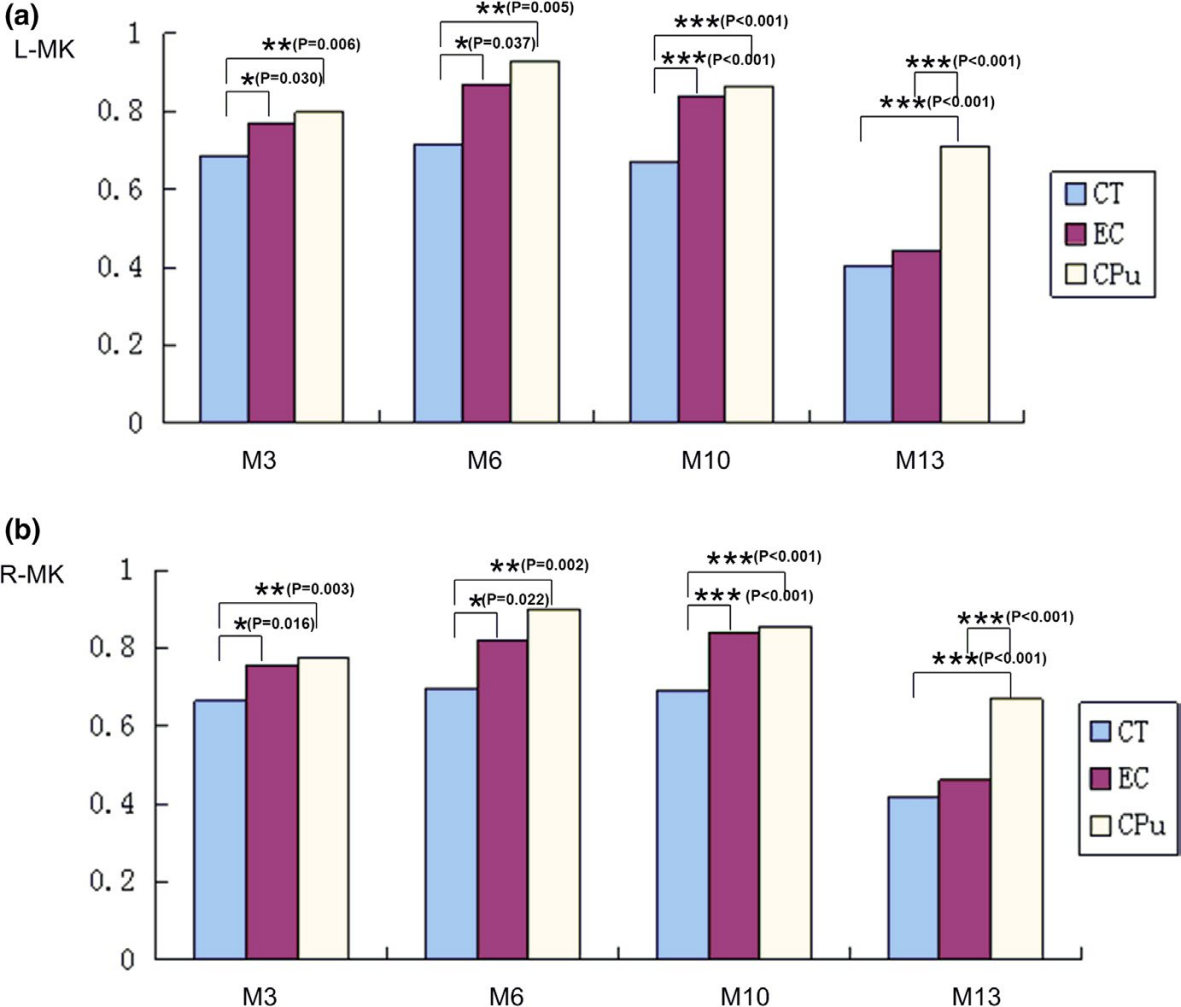
Comparison of the MK values at M3, M6, M10, and M13 among different brain regions. *p < .05; **p < .01

The results of measurement and statistical analysis of the MK values for M3, M6, M10, and M13 age groups at each brain region are summarized in Figure 4. It was found that MK values for M3, M6, and M10 age groups vary across the three brain regions examined, whereas the MK value for M13 group was only significantly different between the CPu and both the CT and the EC.

**FIGURE 5:**
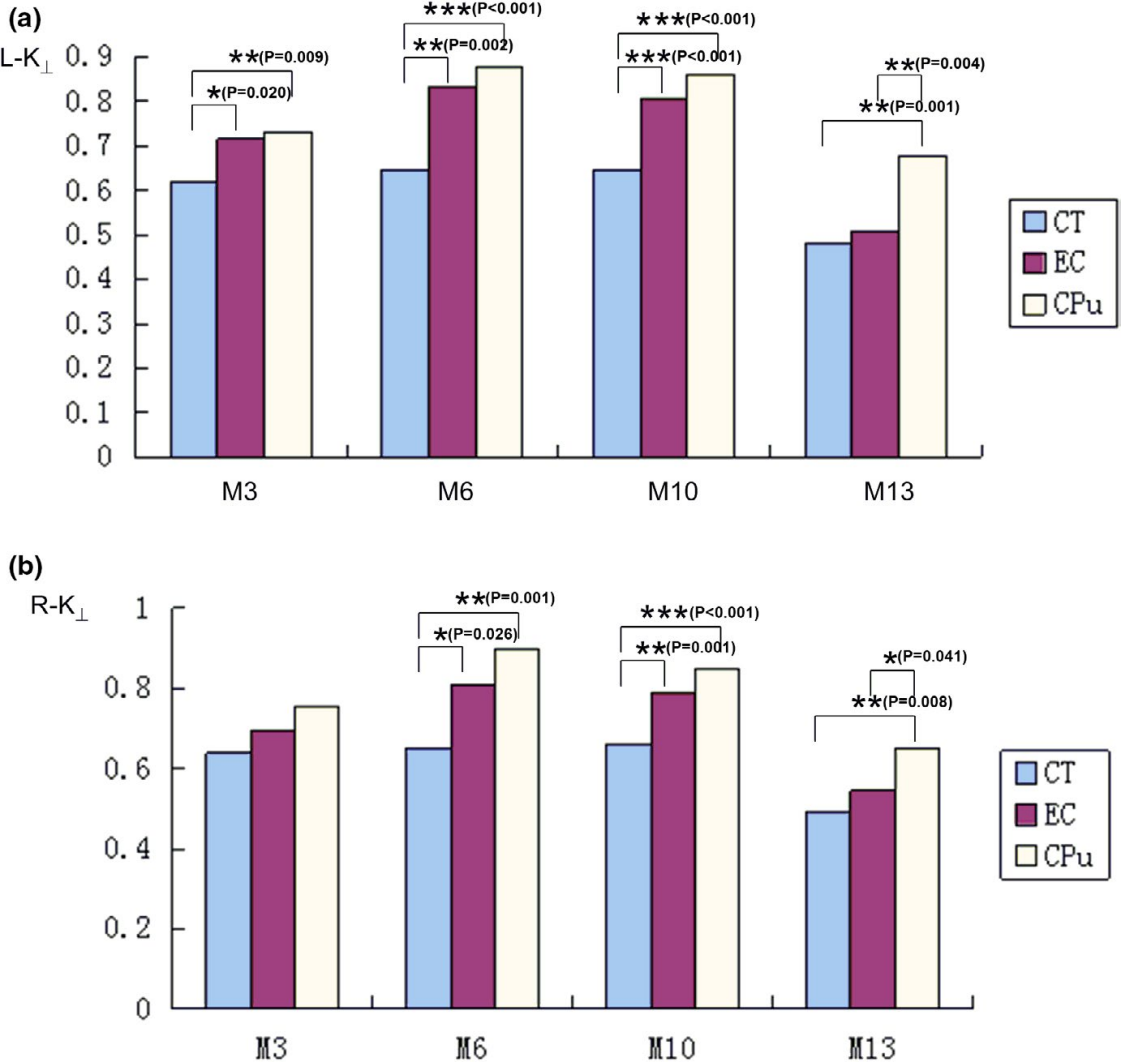
Comparison of the K_⊥_ values at M3, M6, M10, and M13 among different brain regions. *p < .05; **p < .01

The results of measurement and statistical analysis of the K_⊥_ values at M3, M6, M10, and M13 age groups for each brain region are summarized in Figure 5. No significant difference in the K_⊥_ values was observed among the examined brain regions in the left hemisphere for M3 age group. In contrast, significant differences in the K_⊥_ values were observed between the CT and both the EC and the CPu in the right hemisphere for M3 age group in both hemispheres at M6 and M10. Lastly, the K_⊥_ value at the CPu region was different from that for the CT and the EC for M13 age group.

The average MK and K_⊥_ values at M3, M6, M10, and M13 varied from largest to smallest in the following order: CPu>EC>CT.

The scatter diagrams of the MK and K_⊥_ values for each brain region over time showed a nonlinear relationship. To further evaluate the correlation between the diffusion values and age groups, four models, exponential, logarithmic, quadratic, and cubic models were assessed for curve fitting of the MK and K_⊥_ values. The results showed that the MK and K_⊥_ values of the CT, the EC, and the CPu in both hemispheres over time can best fit a parabolic curve, the peak of which corresponded to an age of approximately 6 months.

The quadratic functions of the MK and K_⊥_ values for the CT, the EC, and the CPu in both hemispheres were as follows:

MK

$$\text{Left CT}:Y=0.477+0.086X‐0.007X2\left(R^{2}=.57,p=.000\right),$$

$$\text{Right CT}:Y=0.426+0.096X‐0.007X2\left(R^{2}=.599,p=.000\right),$$

$$\text{Left EC}:Y=0.382+0.159X‐0.012X2\left(R^{2}=.712,p=.000\right),$$

$$\text{Right EC}:Y=0.387+0.146X‐0.011X2\left(R^{2}=.688,p=.000\right),$$

$$\text{Left CPu}:Y=0.565+0.099X‐0.007X2\left(R^{2}=.351,p=.000\right),\;\text{and}$$

$$\text{Right CPu}:Y=0.524+0.106X‐0.007X2\left(R^{2}=.365,R^{2}=.000\right)$$

K_⊥_

$$\text{Left CT}:Y=0.486+0.054X‐0.004X2\left(R^{2}=.307,R^{2}=.001\right),$$

$$\text{Right CT}:Y=0.515+0.05X‐0.004X2\left(R^{2}=.282,R^{2}=.002\right),$$

$$\text{Left EC}:Y=0.394+0.133X‐0.01X2\left(R^{2}=.675,R^{2}=.000\right),$$

$$\text{Right EC}:Y=0.388+0.126X‐0.009X2\left(R^{2}=.547,R^{2}=.000\right),$$

$$\text{Left CPu}:Y=0.473+0.108X‐0.007X2\left(R^{2}=.321,R^{2}=.001\right),\;\text{and}$$

$$\text{Right CPu}:Y=0.467+0.12X‐0.008X2\left(R^{2}=.337,R^{2}=.001\right).$$

## 4 DISCUSSION

The focus of this study was to explore the age‐related changes in *SD* rats by taking advantage of the Diffusion Kurtosis Imaging technique and analyzing the tissue diffusion values. This study only included male rats in order to avoid the potential effect from estrogen and different structure from different gender. Future work including female remain further explored.

### 4.1 Comparing the age of rats to the age of humans

A comprehensive comparison of rat age versus human age was described in a previous article: sexual maturity occurs in rats at about six weeks of age and in humans at approximately 12 to 13 years of age (Andreollo et al., 2012). Social maturity is attained at five to six months of age in rats and between 18 and 20 years in humans. A rat at 12 months old is equivalent to a human at 35 to 40 years old, and a 24‐month‐old rat is equivalent to a 65‐ to 70‐year‐old human. This study used *SD* rats of three, six, ten, and thirteen months old, which are equivalent to humans at the stages of development, maturity, and aging.

### 4.2 The laterality of the CT, the EC, and the CPu for each parameter

The left and right cerebral hemispheres are unified, but their functions are not exactly identical. One study found that the left and right hemispheres were asymmetrical in terms of both structure and function; specifically, the cerebral volume and the gray matter to white matter ratio of the left hemisphere exceed those of the right hemisphere (Takao et al., 2011). To determine whether diffusion values displayed laterality, this study analyzed the laterality of the MK and K_⊥_ values for multiple brain regions. The results indicated no laterality of the MK and K_⊥_ values at the EC, CT, or CPu regions. One previous voxel‐based DTI analysis of the whole brain by Inano S and colleagues also found no significant difference in diffusion values between hemispheres, and the lack of laterality does not change with age (Inano et al., 2011).

Both MK and K_⊥_ values reflect the diffusion properties of water molecules within a structure. Therefore, the factors that affect the diffusion of water molecules can alter the MK and K_⊥_ values. One study reported no significant laterality in the total volume of cerebral white matter, the total volume of myelinated nerve fibers in white matter, the total length, length density, volume density or average diameter of myelinated nerve fibers, or the number of oligodendroglia (Inano et al., 2011). In addition, that study demonstrated that the number of neurons in the cortex did not decrease appreciably over time (Lin, 2009). All of the above results can effectively explain why no significant difference in diffusion parameters was found between the left and right cerebral hemispheres.

### 4.3 Comparison of the diffusion parameters between age groups and their correlation with age

In this study, the MK values for the CT, the EC, and the CPu in both hemispheres differed among age groups, although not between every two age groups based on our studies have covered test. In particular, a greater difference in the MK values for different brain regions was found between 13 months and the rest age groups. MK can be regarded as an index of tissue microstructural complexity. The results showing a difference in MK values between age groups emphasize that the organizational microstructure of the brain is continually developing. Age‐related analysis and curve fitting revealed that the MK values for all examined brain regions first positively correlated and then negatively correlated with age, fitting a parabolic function. Cheung et al. (2009) reported that MK in rodents gradually increased from birth to maturity, as reflected by increasing region of the parabola. Alternatively, the downward stage corresponded to the results of Lätt et al. (2013) Regarding the subjects examined, Cheung et al. selected normal *SD* rats of 3 different ages: postnatal days 13, 31, and 120; these time points extended to only maturity. In contrast, Lätt et al. exclusively examined normal adults greater than 20 years old as subjects. By comparison, the present study combined the design of the above two reports. Thus, our results provide a more accurate and comprehensive description of the development of normal brain tissues.

From the physiological perspective, the period from birth to maturity is an important stage of brain tissue development. White matter maturation processes include increasing the density of fiber bundles and axons, the diameter of axons and the number of neurofibrils, altering axonal membrane permeability (Dubois et al., 2006; Hüppi & Dubois, 2006; Larvaron et al., 2007; Neil et al., 2002; Suzuki et al., 2003), and enhancing the complexity of the extracellular matrix and microtubule‐associated proteins (Hüppi & Dubois, 2006; Neil et al., 2002; Suzuki et al., 2003). In gray matter, aside from the addition of basal dendrites and the modification of tissue water content and cell packing density, changes in cortical cytoarchitecture are known to affect water diffusion behavior (Bockhorst et al., 2008; Hüppi & Dubois, 2006; Sizonenko et al., 2007). Therefore, the MK values positively correlate with age during this stage. With further increases in age, nerve fibers become damaged, myelin breaks down and the total length, number of nodes, number of dendritic spines, and density of dendritic spines decrease, all of which reduce the impedance of water diffusion (Duan et al., 2003; Page et al., 2002). Therefore, the MK values negatively correlate with age at this stage.

Identical to MK, K_⊥_ displayed parabolic changes against age. A possible explanation for this finding is that K_⊥_ is related to the integrity of nerve fibers and during the stages of brain development and maturity, the K_⊥_ values increase due to the processes of myelination and dense axon fiber packing, which greatly restrict diffusion in the radial direction. The subsequent decrease in K_⊥_ results from myelinoclasis and debris formation, which reduce the impedance of water diffusion. As for MK, which was affected by age, K_⊥_ initially increased in a pattern that was identical to the results of Cheung et al. and then decreased in a manner that was identical to the results of Lätt et al. In pathological cases, in which myelin breakdown and debris formation are observed, K_⊥_ will decrease. As the K_⊥_ value reflects the integrity of nerve fibers in a structure, many clinical investigators have used K_⊥_ to evaluate a variety of central nervous system diseases, especially leukodystrophy, aiming to more rapidly detect demyelinating diseases.

### 4.4 Comparison of the MK and K⊥ values among different brain regions

The magnitude of the mean MK and K_⊥_ values displayed the following pattern from largest to smallest: CPu>EC>CT. Because MK is regarded as an index of tissue microstructural complexity, based on these results, we propose that the structure of the CPu is the most complex, followed by the EC and, finally, the CT. The authors hypothesize that these differences are related to the structural characteristics of these three organizational structures. The EC, a white matter tissue, is composed of neurites encapsulated by myelin into myelinated nerve fibers. Circular phospholipids of myelin sheath, the outer layer of nerve fibers, clearly limit the diffusion of water molecules. The CT is a gray matter structure that is composed of the cell bodies of neurons. The CPu contains a complex mixture of gray matter and white matter; thus, the properties of water molecule diffusion in the CPu are affected by the diffusion properties of both gray matter and white matter.

## 5 CONCLUSION

In conclusion, we have studied the changes of the water diffusion values in different brain regions among different age groups based on DKI results using rodents model. We subsequently determined the correlations of these values with age, and plotted these values in various brain regions against age groups. This outcome has covered a relative spectrum of subject age groups and are complementary to the existing studies. DKI has been used as an efficient tool for evaluating brain aging process and this method will lay the foundation and provide new concepts for further studies of normal brain aging and neurodegenerative diseases. However, due to the limited numbers of age groups and experimental subjects, the observation of significant results is yet to be further supported by more extensive experimentation. Selecting a longitudinal study design would yield additional information about brain aging. Study of greater numbers of subjects and age groups will enable us to obtain equation coefficients for improved accuracy and produce more reliable conclusions. With the wide application of DKI in scientific research and clinical practice, we believe that DKI will facilitate the study of normal aging‐related and pathological changes in brain tissue.

## CONFLICT OF INTERESTS

The authors declare that they have no competing interests.

## AUTHORS’ CONTRIBUTIONS

Xuefang Han: study design, data acquisition and analysis, manuscript drafting and revising; Zuojun Geng: study design, data analysis, manuscript drafting and revising; Qingfeng Zhu and Zhenhu Song: study design, data acquisition, and manuscript revising; Huandi Lv: study design, data acquisition, manuscript drafting and revising. All authors read and approved the final manuscript.

### PEER REVIEW

The peer review history for this article is available at https://publons.com/publon/10.1002/brb3.2136.

## Data Availability

The datasets generated and analyzed during the current study are available from the corresponding author on reasonable request.
